# Impact of combined acidic and oxidative stress on guanine DNA damage

**DOI:** 10.1038/s41598-026-67593-9

**Published:** 2026-08-27

**Authors:** Islam G. Ali, Nada A. Khaled, Hanan Elhaes, Medhat A. Ibrahim

**Affiliations:** 1 Physics Department, Faculty of Science, Arish University, Arish, 45511 Egypt; 2 Therapeutic Chemistry Department, National Research Centre, 33 El-Bohouth St., Dokki, Giza, 12622 Egypt; 3 Physics Department, Faculty of Women for Arts, Science and Education, Ain Shams University, Cairo, 11757 Egypt; 4 Spectroscopy Department, National Research Centre, 33 El-Bohouth St., 12622, Dokki, Giza, Egypt; 5 Center for Converging Sciences and Emerging Technologies (CoSET), Benha National University (BNU), El-Obour, 13518 Egypt; 6 Molecular Modeling and Spectroscopy Laboratory, Centre of Excellence for Advanced Science, National Research Centre, 33 El-Bohouth St., 12622, Dokki, Giza, Egypt

**Keywords:** DNA damage, Guanine, DFT, HOMO–LUMO, MESP QTAIM, NBO and NCI, Biochemistry, Biophysics, Chemical biology, Chemistry

## Abstract

This research investigates guanine reactivity through quantum-chemical methods which study its behaviour during simultaneous oxidative and acidic stress using Density Functional Theory at the B3LYP/6-31G(d, p) computational method. The researchers tested three systems which contained the •OH group addition at C17 (canonical C8) and the H_3_O^+^ group protonation at N16 (canonical N7) and the combination of •OH and H_3_O^+^ attack. The chemical stability assessment uses HOMO–LUMO energy gap and Molecular Electrostatic Potential (MESP) evaluation to identify reactive sites which emerge because of stress. The topological assessment through QTAIM and NCI shows that •OH creates a C–O bond with C17 through covalent bonding which results in π-conjugation loss while H_3_O^+^ mostly protonates N16 to create a hydrogen-bonded structure. The NBO second-order perturbation analysis shows that phosphate oxygens together with their neighboring lone pairs produce strong hyperconjugative (n →σ*) interactions which help stabilize both single and dual damage. The analysis of HOMO–LUMO shows how electronic states get redistributed while MESP maps display changes to guanine’s electrophilic and nucleophilic areas that occur during simultaneous attacks. The dual-attack ensemble creates maximum electron-density redistribution which causes strong steric strain that produces more biological persistence and affects how DNA repair systems recognize it. The study shows how guanine gets damaged in acidic environments which have elevated ROS levels because these conditions result in both mutagenesis and radiobiological effects.

## Introduction

DNA damage is a central driver of radiation-induced biological effects, including cancer cell death, aging, and therapeutic resistance; the accumulation of unrepaired damage destabilizes the genome and fosters oncogenesis^1^. In particular, oxidative DNA damage caused by reactive oxygen species (ROS) account for the majority of endogenous damage^2,3^. Among the four nucleobases, guanine (G) is especially susceptible: it has the lowest one-electron oxidation potential (≈ 1.29 V vs. normal hydrogen electrode (NHE)) of all bases^4^. Consequently, hydroxyl radicals (•OH) – generated by ionizing radiation or Fenton chemistry in cells – attack DNA and preferentially oxidize guanine^5^. These attacks often abstract an electron (or H-atom) from G, initiating products like 8-oxo-7,8-dihydroguanine (8-oxoG). 8-oxoG is a potent mutagenic intermediate and a well-established biomarker of oxidative stress^6,7^. Indeed, 8-oxoG accumulation is considered a hallmark of chronic ROS exposure and has been directly implicated in cancer development^6^. Thus, ROS-mediated guanine oxidation is a key mechanistic link between cellular metabolism, DNA mutagenesis, and tumorigenesis. Tumors and inflamed tissues often present acidic microenvironments (extracellular pH ~ 6.5–6.9) due to hypoxia and high glycolytic metabolism^8,9^. This pathological acidosis increases the local proton (H₃O⁺) concentration around DNA. Guanine’s chemical structure makes it highly prone to protonation under such conditions: the N7 nitrogen of G has the highest proton affinity among all nucleobases^10^. Protonation of guanine (primarily at N7) reconfigures its charge distribution and hydrogen-bonding capacity^10^. Despite extensive study of ROS or low-pH damage in isolation, the combined effect of oxidative and acidic stress on DNA is poorly understood. In other fields it is known that ROS plus acid can act synergistically: e.g. in innate immunity, phagolysosomes use a cocktail of reactive oxygen and nitrogen species (RONS) in an acidic milieu to efficiently kill microbes^11,12^. By analogy, one expects that flooding DNA with H₃O⁺ in the presence of ROS might modulate the damage outcome – for example, by changing reaction pathways, damage yields or repairability. However, most prior work has focused on either ROS-induced damage (e.g. 8-oxoG formation by •OH)^4,6,13–15^ or acid-driven protonation (e.g. pH-dependent base modifications)^16^ separately. A unified biophysical framework that quantifies guanine’s reactivity under both oxidative radicals and hydronium ions is lacking. This gap hampers predictive models of DNA mutagenesis and resistance in complex pathophysiological conditions. Molecular modeling represents a versatile class of computational techniques that rely on quantum chemical methods, such as Density Functional Theory (DFT), to systematically investigate the electronic structure and functional properties of complex systems, often complementing experimental spectroscopic analyses^17^. In biological contexts, these methods play a pivotal role in both designing applications and elucidating underlying mechanisms^18,19^. Computational approaches have been widely employed to evaluate key electronic and physical parameters, including the HOMO–LUMO energy gap (ΔE) and Total Dipole Moment (TDM), across diverse systems such as biopolymers and natural nanocomposites. Such studies also extend to material functionalization strategies aimed at improving their performance in biomedical applications^20–23^. Beyond materials science, molecular modeling has been applied to address a wide spectrum of problems in chemistry, biology, and physics, frequently in combination with complementary tools such as Quantitative Structure-Activity Relationship (QSAR) modeling and molecular docking^24,25^. Computational chemistry offers a powerful framework to bridge the gap between experimental observations and mechanistic understanding. In particular, DFT provides molecular-level insights into electronic structure changes, such as those occurring in guanine under simultaneous oxidative and acidic stress, thereby illuminating its reactivity^26^. Advanced analyses, including Quantum Theory of Atoms in Molecules (QTAIM) for mapping bond critical points and electron density redistribution, Natural Bond Orbital (NBO) analysis for clarifying charge transfer and donor–acceptor interactions^27,28^, and Non-Covalent Interaction (NCI) analysis for visualizing stabilizing or destabilizing weak interactions^29^, enable a rigorous interpretation of complex molecular behavior. Together, these approaches offer a comprehensive picture of structural and electronic responses under multifactorial stress, complementing experimental findings and strengthening predictive models of DNA damage. In this study, we employ DFT-based analysis to investigate guanine oxidation by hydroxyl radicals (•OH at C8), protonation by hydronium ions (H₃O⁺ at N7), and their simultaneous action. We analyze how these stressors influence guanine’s structure, stability, and electronic properties. QTAIM characterizes bond topologies and electron density redistribution, NBO highlights charge-transfer pathways, and NCI reveals altered hydrogen bonding and van der Waals contacts under dual attack. Our central hypothesis is that combined oxidative and acidic stress substantially modifies guanine’s reactivity compared to either condition alone. By quantifying charge redistribution, bonding changes, and non-covalent interactions. The present computational findings provide biophysical, molecular-level insights into how concurrent oxidative (•OH at C8) and acidic (H₃O⁺ at N7) stressors alter the electronic landscape of the guanine nucleotide. To characterize these nucleobase-level alterations, we apply DFT, QTAIM, and NBO analyses to evaluate structural distortions and electronic rearrangements. The HOMO–LUMO gap serves as a quantitative stability indicator, while Molecular Electrostatic Potential (MESP) surfaces localize reactive sites susceptible to electrophilic or nucleophilic attack. Topological and NCI analyses further clarify donor–acceptor interactions and secondary stabilization contributions from the DNA backbone. Together, these analyses detail the intramolecular factors governing the physicochemical behavior of complex damage under simultaneous stressors, within the framework of the present computational model.

## Computational details

**Fig. 1:**
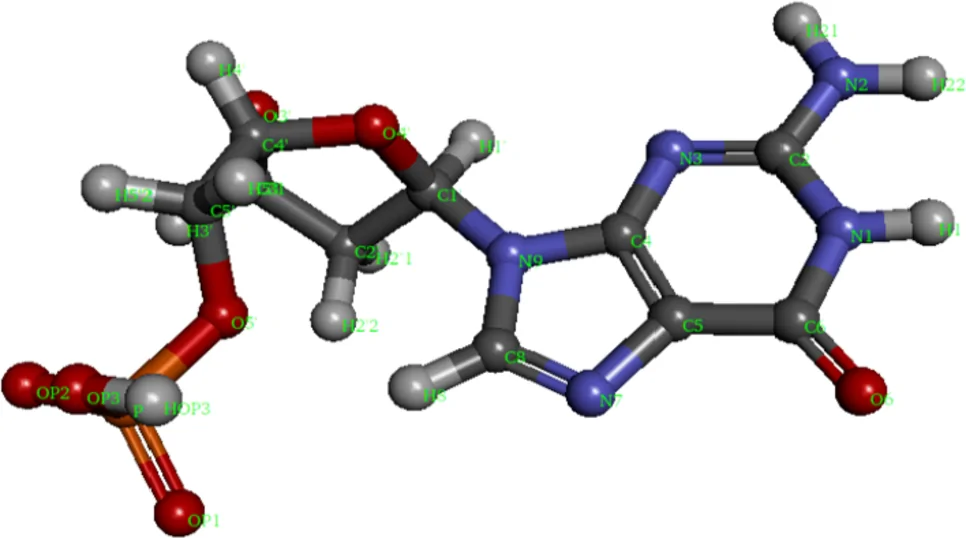
Neutral guanine fragment extracted from an ideal B-DNA duplex using BIOVIA Discovery Studio 2021. The gas-phase model preserves the crystal geometry, with atoms labeled: blue (N), black (C), red (O), and gray (H)^30^.

**Fig. 2:**
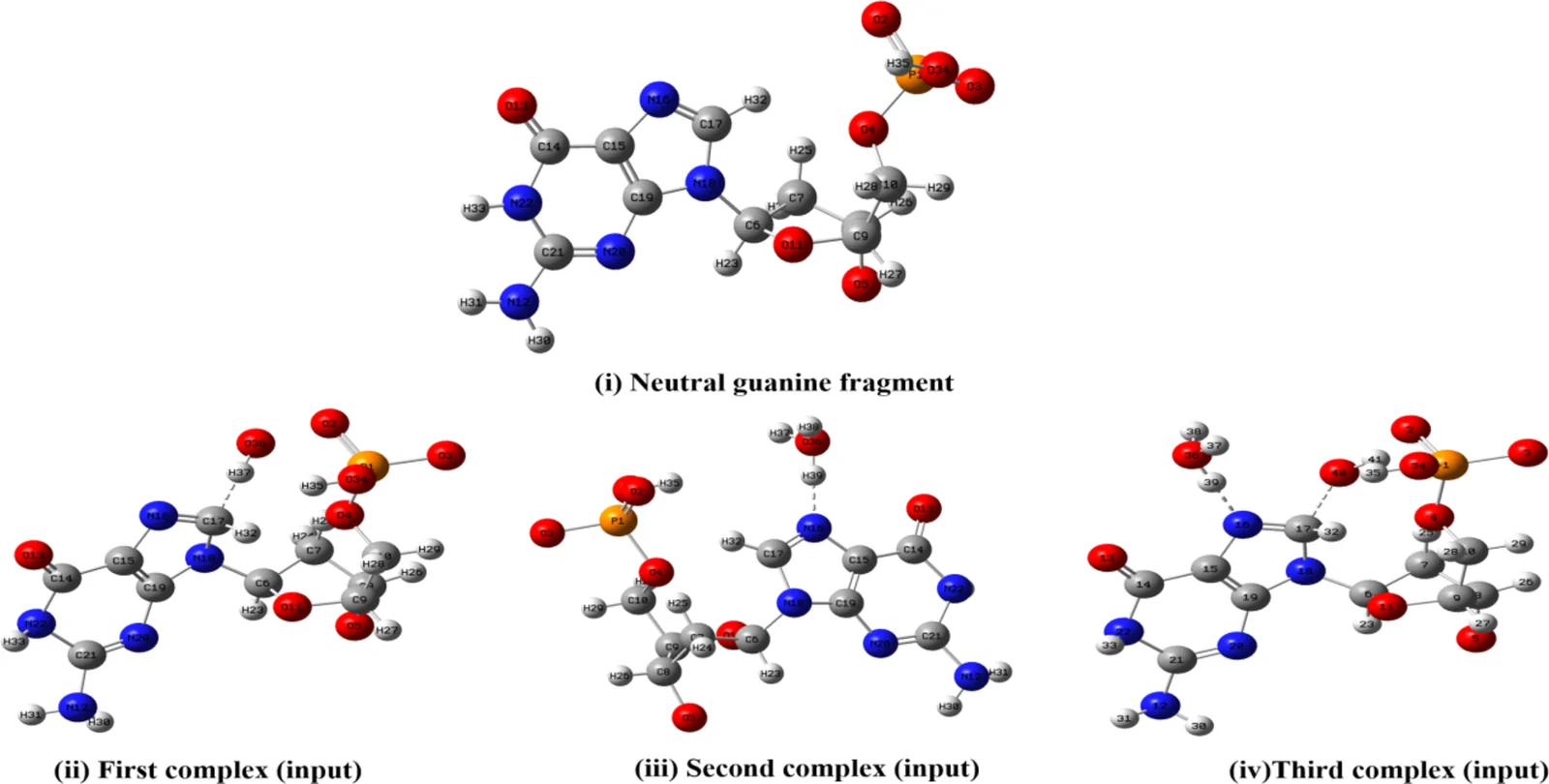
Input structures: (i) neutral guanine, (ii) guanine–•OH complex with attack at C17, (iii) guanine–H₃O⁺ complex with attack at N16, and (iv) guanine complex with simultaneous •OH and H₃O⁺ attacks at C17 and N16, respectively.

A multiscale computational workflow was employed to investigate the reactivity of guanine nucleotide under combined oxidative (•OH) and acidic (H₃O⁺) stress conditions in DNA. molecular model is a guanine-5’-monophosphate fragment (guanine base + deoxyribose + phosphate) extracted from an idealized B-DNA duplex, corresponding to a single-nucleotide DNA fragment using BIOVIA Discovery Studio 2021^30^(Fig. 1) and modeled in the gas phase as an electronically neutral system while preserving the atomic coordinates from the original B-DNA geometry. All quantum chemical calculations were performed using the Gaussian 09 W software package^31^. Initial geometry optimization of the guanine fragment was conducted at the B3LYP/6-31G(d, p)^32^ level of theory. Following structural optimization, comprehensive electronic structure analyses were performed using the quantum theory of atoms in molecules (QTAIM), natural bond orbital (NBO), and noncovalent interaction (NCI) methodologies to identify potential reactive sites and characterize intermolecular interactions. Three representative reaction pathways were systematically explored: (i) hydroxyl radical (•OH) attack at the C8 position of guanine (designated as atom C17 in the computational model), (ii) hydronium ion (H₃O⁺) attack at the N7 position (designated as atom N16), and (iii) simultaneous •OH and H₃O⁺ attacks at positions C8 and N7, respectively (Fig. 2). For each reaction pathway, the resulting molecular complexes were subjected to full geometry optimization at the B3LYP/6-31G(d, p) level, HOMO/LUMO and MESP were calculated at the same level of theory followed by comprehensive QTAIM, NBO, and NCI analyses to elucidate the electronic characteristics and reactive interactions. QTAIM and NCI calculations were performed using the Multiwfn program suite^33^, while NBO analyses were conducted using NBO 3.1^34^ as implemented in Gaussian 09 W. Molecular visualization and figure preparation were accomplished using GaussView 6^35^, VMD (Visual Molecular Dynamics)^36^, and Gnuplot^37^ to generate publication-quality molecular graphics and data plots. To ensure consistency between computational results and structural representations, the internal DFT atom indices were retained throughout all figures and analyses. Specifically, the computational atom label C17 corresponds to the canonical C8 position of guanine according to IUPAC nomenclature, while N16 corresponds to the canonical N7 position. This labeling convention maintains direct correspondence between structural depictions, coordinate data, and supplementary materials, thereby facilitating reproducibility and interpretative clarity.

## Results and discussion

### Geometry optimization and electronic structure

**Fig. 3:**
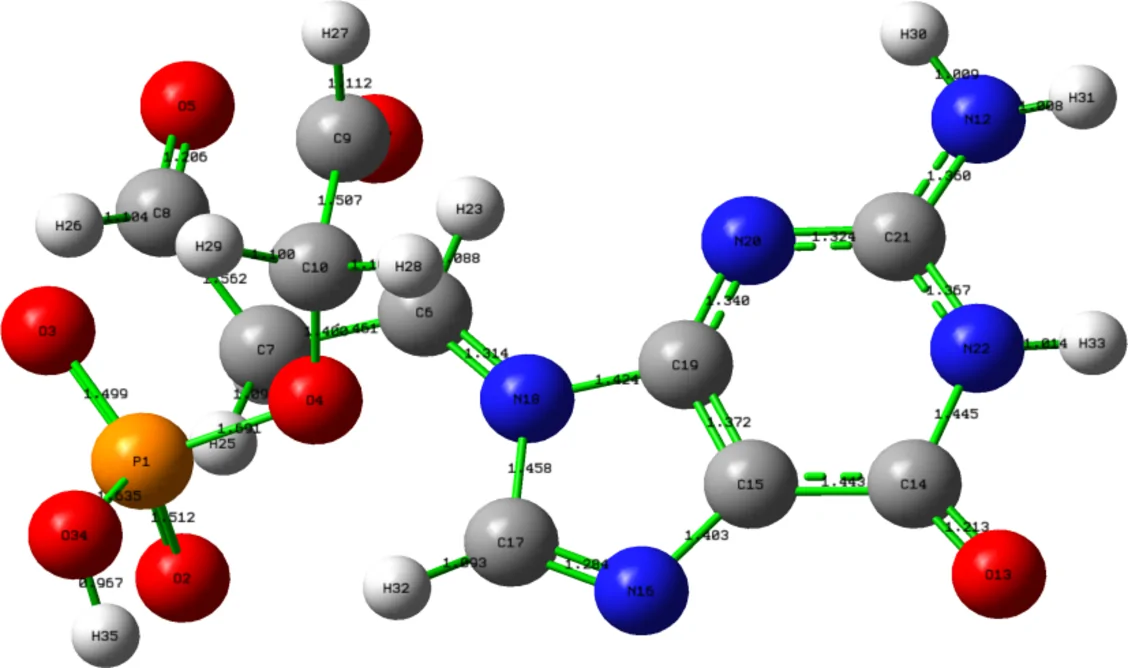
Optimized geometry of neutral guanine at the B3LYP/6-31G(d, p) level. Atom labels correspond to internal DFT indices (see Computational Details).

The guanine single-strand structure was fully optimized at the B3LYP/6-31G(d, p) level of theory without symmetry restrictions. The optimized geometry (Fig. 3) shows overall planarity of the purine ring system, consistent with the aromatic character of nucleobases. Characteristic bond lengths fall within the expected range: the C = O bond of the pyrimidine ring is 1.213 Å, while the C–N bonds within the ring (1.324, 1.340, and 1.367 Å) exhibit partial double-bond character due to π-conjugation and resonance stabilization. The aromatic C = C bonds measure 1.372 and 1.443 Å, confirming delocalization of π-electrons across the heteroaromatic framework. For the phosphate backbone, P–O single bonds were calculated at ~ 1.635 Å, whereas the P = O bond is shorter (1.499 Å), reflecting the expected double-bond nature. These structural parameters align well with reported crystallographic and theoretical values for guanine derivatives, validating the computational approach [38]. Importantly, the delocalized π-system of the aromatic carbons enhances their susceptibility to electrophilic attack by hydroxyl radicals, whereas hetero-nitrogens represent secondary nucleophilic sites. Such optimized electronic distribution provides the foundation for subsequent QTAIM and NCI analyses, which further elucidate interaction pathways and spectroscopic signatures of guanine under oxidative and acidic stress. We note that the extracted fragment retains the anti-conformation about the glycosidic bond (C1′–N9), characteristic of the idealized B-DNA duplex. In B-DNA, the guanine nucleotide can adopt a syn conformation, which influences frontier orbital character: in the anti-conformation the π HOMO may localize on the phosphate group, while in the syn conformation near-degenerate base-centered and phosphate-centered ionizing states arise [39]. The present calculations reflect the anti-conformer structure.

### Calculated HOMO/LUMO and MESP

**Fig. 4:**
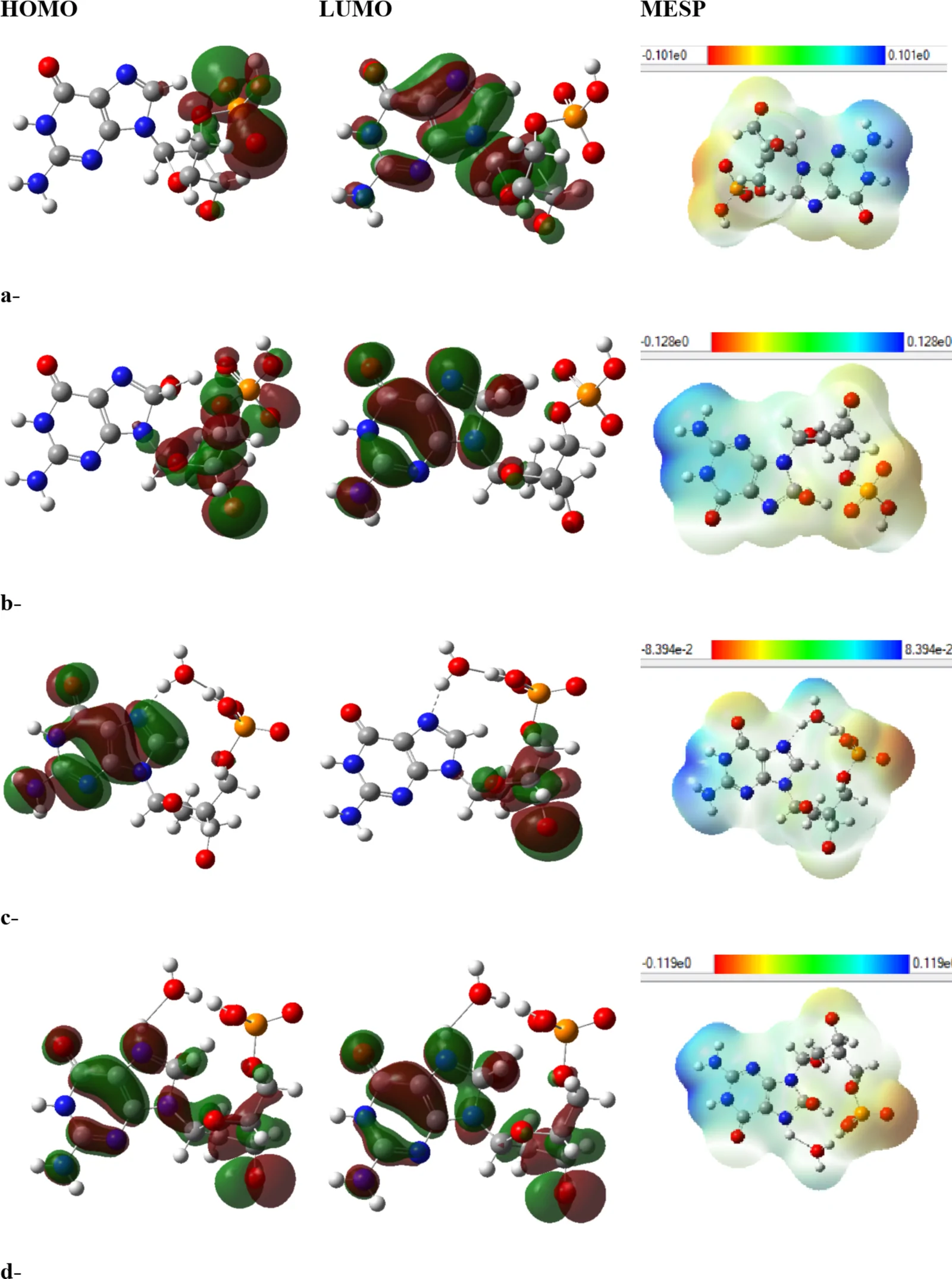
MESP, HOMO and LUMO for the studied structures whereas (a) neutral guanine, (b) guanine–•OH complex with attack at C17, (c) guanine–H₃O⁺ complex with attack at N16, and (d) guanine complex with simultaneous •OH and H₃O⁺ attacks at C17 and N16, respectively.

The electronic properties of guanine in its neutral and radical/ion-attacked states are shown in Fig. 4 through its Highest Occupied Molecular Orbital (HOMO) and Lowest Unoccupied Molecular Orbital (LUMO) and Molecular Electrostatic Potential (MESP) measurements. The HOMO represents the regions with the highest electron density, identifying where the molecule is most likely to act as an electron donor (nucleophile). The MESP maps show red or orange zones as the areas which display negative electrostatic potential, MESP analysis follows the conceptual framework of Suresh et al. [40] and Gadre et al. [41] for interpreting chemical reactivity. The HOMO in neutral guanine together with its complexes shows its main distribution over the nucleobase and certain functional groups (like the phosphate group in certain configurations), which corresponds to the red-coded areas of the MESP that indicate electron-rich regions which attract electrophiles. The LUMO shows which parts of the system can take in electrons while demonstrating which sections of the molecule most easily become targets for nucleophilic attacks. The MESP map shows these areas through its blue or dark green zones which indicate areas of positive electrostatic potential that present electron deficiency. As in Fig. 4, the LUMO density often shifts depending on the attacking species (OH or H_3_O^+^). For instance, in the H_3_O^+^ complex (c), the positive charge of the hydronium ion influences the MESP to show more blue/positive regions, correlating with the shift in LUMO position toward the site of protonation.

The first figure of 4-a displays the structure of neutral Guanine which maintains equal distribution because the base holds its main reactive sites for both HOMO and LUMO. The second Fig. 4-b shows how hydroxyl radical introduction creates changes in local electron distribution. The MESP shows potential changes at the C17 position which correspond to the rearrangement of HOMO/LUMO lobes around the new bond. The MESP about Fig. 4-c shows that N16 protonation creates a strong positive potential which appears as a blue area in the MESP. The LUMO exhibits a strong relationship with this area because the positively charged region functions as the primary electron acceptance location for upcoming chemical reactions. The fourth figure shows that electronic distribution undergoes its maximum distortion at 4-d. The MESP scale (which extends from − 0.119 to + 0.119) demonstrates that when an oxidant and an acid exist together, they cause chemical active sites to move throughout the entire guanine derivative.

### Electronic structure analysis of neutral guanine via QTAIM

#### Reactive site identification

**Fig. 5:**
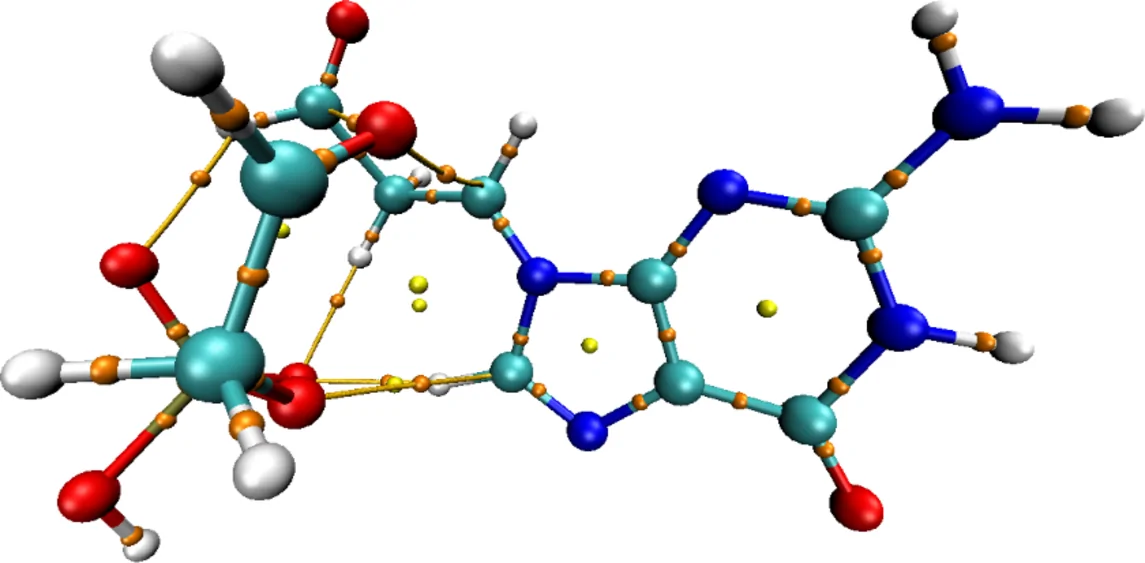
QTAIM analysis of guanine DNA single-strand structure.

The Quantum Theory of Atoms in Molecules (QTAIM) analysis was employed to identify potential hydroxyl radical (•OH) attack sites within the neutral guanine by examining nuclear critical points (NCPs) and associated electronic descriptors. Hydroxyl radicals, as highly electrophilic species, preferentially target regions characterized by low average local ionization energy (ALIE), high electron density (ρ), minimal steric hindrance, and negative Laplacian values (∇²ρ < 0), which indicate charge concentration and nucleophilic character. The QTAIM results (Table S1, Fig. 5) reveal that carbon atoms within aromatic rings represent the primary targets for hydroxyl radical attack, exhibiting ALIE values of approximately 9.9 a.u., which facilitate electrophilic addition reactions. Nitrogen atoms in heterocyclic positions serve as secondary targets with ALIE values around 13.8–13.9 a.u., rendering them susceptible to radical addition or electron abstraction processes. In contrast, oxygen atoms display high ALIE values (~ 18 a.u.) coupled with substantial electron density (ρ ~ 290) and strongly negative Laplacian values, indicating enhanced stability and reduced susceptibility to direct radical attacks. Phosphorus centers exhibit exceptionally high ALIE values (72 a.u.), confirming their chemical inertness toward hydroxyl radical interactions. Additionally, hydrogen atoms bonded to carbon or nitrogen centers with low ALIE values (0.4–0.7 a.u.) are particularly vulnerable to abstraction reactions, leading to the formation of carbon- or nitrogen-centered radical intermediates. These findings establish a clear reactivity hierarchy where aromatic carbons constitute the most probable sites for initial hydroxyl radical damage, followed by heterocyclic nitrogens, while oxygen and phosphorus atoms remain relatively protected under normal oxidative conditions.

#### Bond critical point analysis of neutral guanine

Bond critical point (BCP) analysis was performed to identify intramolecular non-covalent interactions within the guanine DNA single-strand structure prior to hydroxyl radical attack. The QTAIM analysis revealed six significant non-covalent interactions (Table S2), comprising three weak hydrogen bonds [CP46: O(2)-H(32), CP58: O(2)-H(25), CP68: O(3)-H(26)] and three weak van der Waals interactions [CP45: O(4)-C(17), CP71: O(11)-C(6), CP76: O(11)-C(8)]. The hydrogen bonding interactions exhibit electron densities (ρ) of 0.020–0.028 a.u. with negative total energy densities (H(r) ≈ -0.001 a.u.), indicating stabilizing interactions and low ellipticity values (ε = 0.011–0.058). The van der Waals interactions display lower electron densities (ρ = 0.008–0.020 a.u.) and slightly positive energy densities (H(r) = 0.001 a.u.), with CP76 showing exceptionally high ellipticity (ε = 2.040) suggesting potential instability. All interactions exhibit positive Laplacian values (∇²ρ = 0.029–0.077 a.u.), confirming their non-covalent nature and contributing to the structural stability that may influence hydroxyl radical attack site accessibility.

### Non-covalent interactions analysis of neutral guanine

**Fig. 6:**
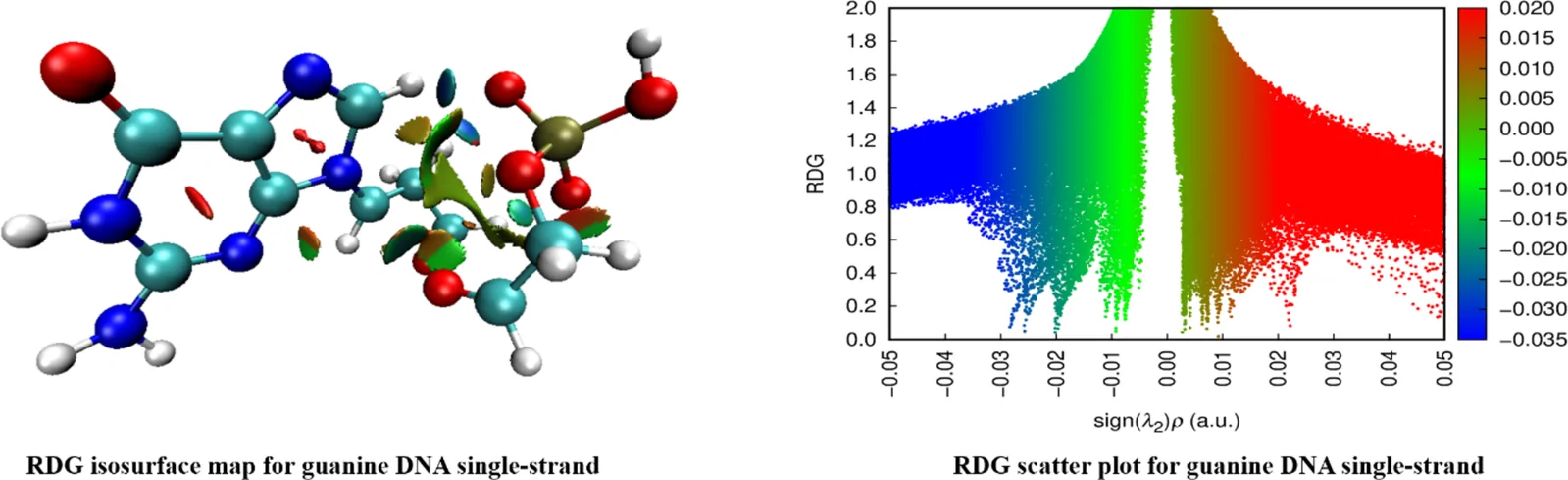
Non-covalent interaction (NCI) analysis of the guanine DNA single strand. Color-coded isosurfaces of the sign(λ₂)ρ function highlight interaction types: blue-hydrogen bonds; green-van der Waals; red-steric repulsion.

Non-covalent interaction (NCI) analysis was employed to visualize weak interactions within the guanine DNA single-strand structure through low-density isosurfaces with reduced density gradients. The sign of the second eigenvalue (λ₂) determines interaction type: negative λ₂ indicates attractive interactions (hydrogen bonding), positive λ₂ corresponds to steric repulsion, and λ₂ ≈ 0 represents weak van der Waals forces. Color-coded isosurfaces display the sign(λ₂)ρ function, where blue regions indicate hydrogen bonds, green areas represent van der Waals interactions, and red corresponds to steric repulsion. The NCI analysis (Fig. 6) corroborates the QTAIM findings, revealing hydrogen bonding and van der Waals interactions in the same regions identified by the bond critical point (BCP) analysis.

### Hydroxyl radical and hydronium ion attack scenarios

**Fig. 7:**
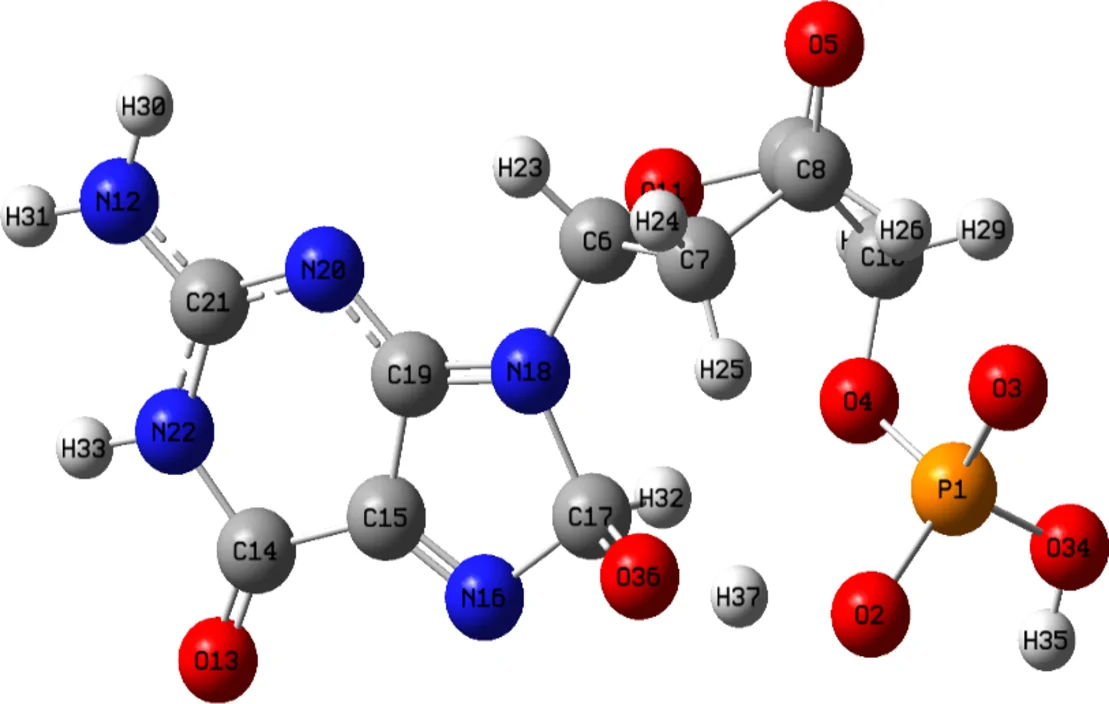
Optimized structure of guanine-•OH complex with attack at C17 (First complex).

**Fig. 8:**
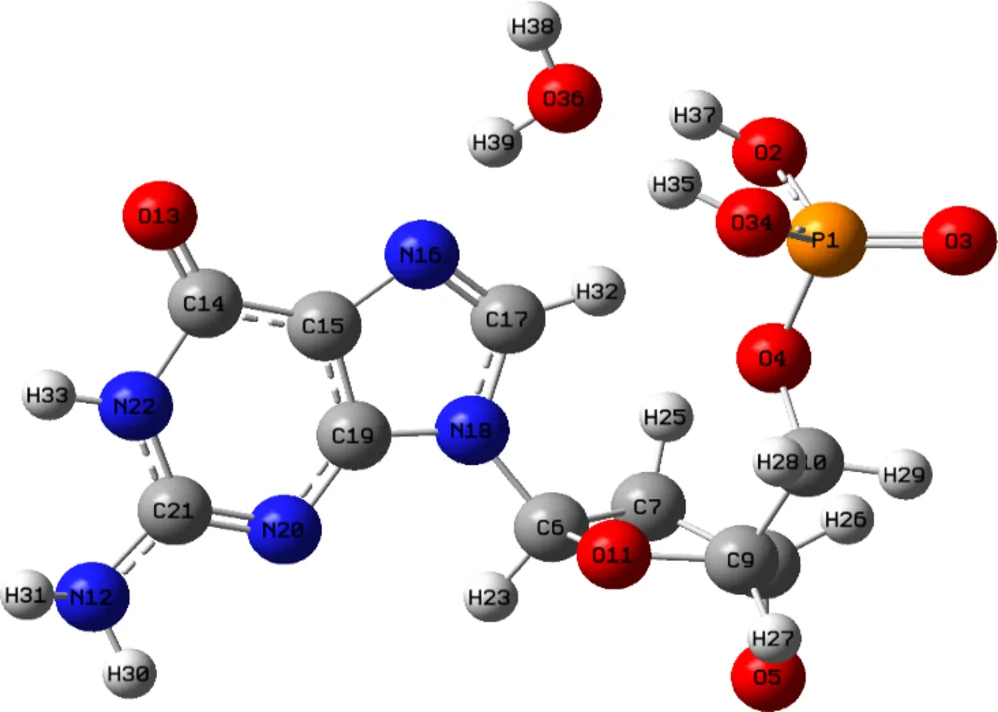
Optimized structure of guanine-H₃O⁺ complex with attack at N16 (Second complex).

**Fig. 9:**
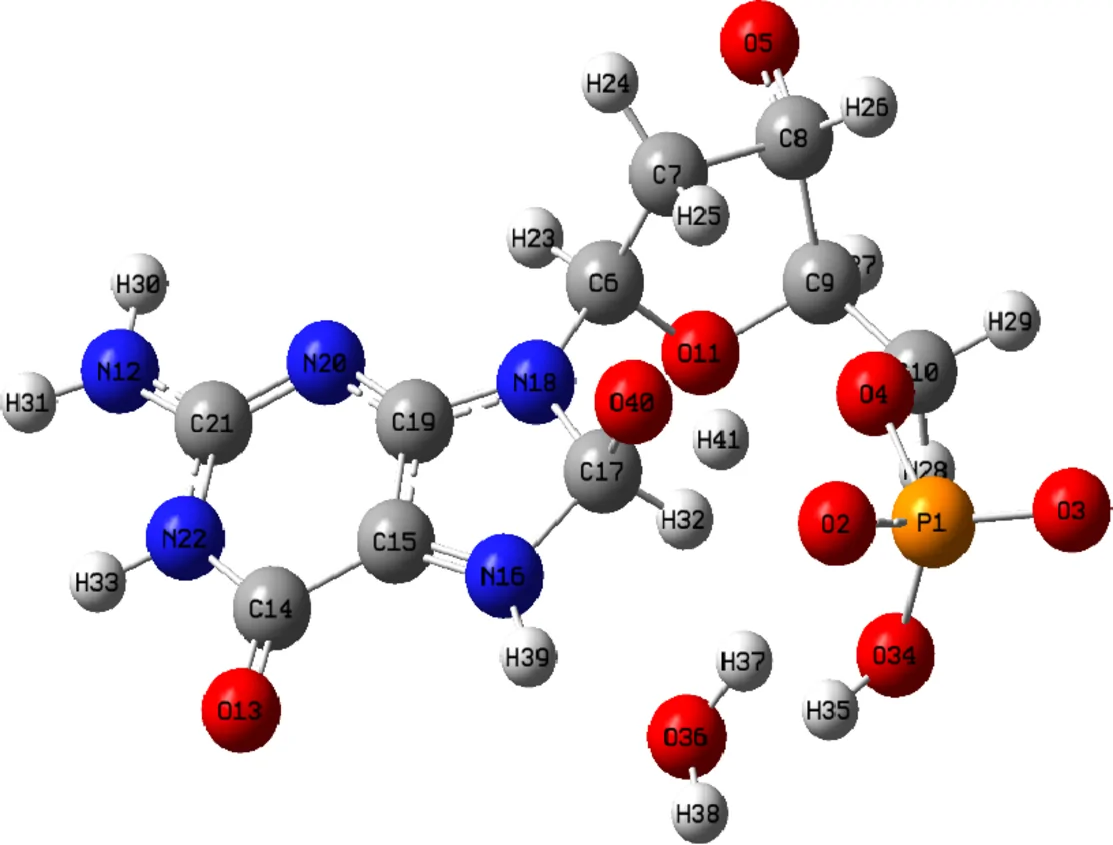
Optimized structure of guanine complex with simultaneous •OH and H₃O⁺ attacks at C17 and N16, respectively (Third complex).

Subsequently, three hydroxyl radical attack scenarios were investigated by optimizing the corresponding complexes structures at the same computational level. The first complex involved guanine DNA single-strand attached by hydroxyl radical (•OH) [O(36)-H(37)] at carbon atom C(17) (Fig. 7). The second complex represented hydronium ion [H(37) O(36)-H(38) H(39)] attack at nitrogen atom N(16) (Fig. 8). The third complex examined dual hydroxyl radical and hydronium ion attacks at both N(16) [H(37) O(36)-H(38) H(39)] and C(17) [O(40)-H(41)], representing a multiple damage scenario (Fig. 9).

#### Quantum reactivity descriptors

To provide a comprehensive quantitative framework for guanine reactivity, a suite of global and local quantum chemical reactivity descriptors was computed for all four systems under investigation—neutral guanine (G), the hydroxyl radical adduct at C17 (G–OH1), the hydronium ion complex at N16 (G–OH31), and the dual-attack system (G–OH1–OH31)—using the frontier molecular orbital energies (Table 1), for neutral guanine displays *ε*HOMO = − 6.3647 eV and *ε*LUMO = − 3.6360 eV, yielding *ΔE* = 2.7288 eV. Upon covalent addition of the hydroxyl radical at C17, the HOMO is marginally stabilized (*ε*HOMO = − 6.4309 eV) while the LUMO deepens considerably (*ε*LUMO = − 4.0213 eV), contracting *ΔE* to 2.4096 eV. This narrowing reflects π-conjugation disruption at C17 following sp²→sp³ rehybridization upon C–O bond formation, in agreement with the QTAIM-confirmed covalent character of the radical adduct (Sect.  3.5.2.1). In contrast, protonation of N16 by the hydronium ion dramatically raises the LUMO to − 0.7377 eV, expanding *ΔE* to 5.4736 eV, the largest gap in the series. This counterintuitive stabilization arises because protonation locks the N7 lone pair into a hydrogen-bonded network, effectively reducing the accessible frontier orbital space and rendering the molecule more resistant to further electrophilic attack under those isolated conditions.

The dual-attack system exhibits the most dramatic electronic reorganization: *ε*HOMO rises to − 5.7767 eV while *ε*LUMO drops to − 4.9389 eV, collapsing *ΔE* to only 0.8378 eV. This near-degenerate frontier orbital manifold signals a profoundly reactive, electronically labile species that is uniquely susceptible to subsequent charge-transfer events, a finding fully consistent with the extensive hydrogen-bonding and covalent network identified by QTAIM and NBO analyses for the dual-stress complex.

Within Koopmans’ theorem approximation [42], the ionization potential (IP = −*ε*HOMO) and electron affinity (EA = −*ε*LUMO) quantify the energetic cost of removing or adding an electron, respectively. Neutral guanine presents IP = 6.3647 eV and EA = 3.6360 eV. Hydroxyl radical addition produces a marginally higher IP (6.4309 eV) and substantially elevated EA (4.0213 eV), indicating an enhanced capacity to accept electrons at the radical-modified carbon locus. The hydronium ion complex retains a comparable IP (6.2113 eV) but a significantly reduced EA (0.7377 eV), reflecting stabilization of the occupied frontier orbital space by protonation while rendering the LUMO energetically inaccessible to further electron capture. The dual-attack adduct achieves the lowest IP (5.7767 eV) in the series, signifying that the concurrent structural and electronic perturbations from both •OH and H₃O⁺ weaken the hold on the outermost electrons and facilitate further oxidative processes. Simultaneously, its EA reaches the maximum value (4.9389 eV), endowing the dual damage with the greatest electron-accepting power and thereby amplifying both oxidative vulnerability and pro-mutagenic potential.

The chemical hardness (*η*) and its inverse, the global softness (*σ*), for Neutral guanine (*η* = 1.3644 eV) and its hydroxyl radical adduct (*η* = 1.2048 eV) display intermediate hardness values, consistent with their ambiphilic character. Protonation at N16 yields the highest hardness (*η* = 2.7368 eV, *σ* = 0.3654 eV⁻¹), leading to charge neutralization by protonation stiffens the electron density distribution and confers kinetic stability against soft electrophilic agents. Conversely, the dual-attack system exhibits the minimum hardness (*η* = 0.4189 eV) and maximum softness (*σ* = 2.3871 eV⁻¹), identifying it as the softest and most polarizable species in the series—an electronic condition that facilitates interaction with a broad range of biological electrophiles and repair enzymes. The Mulliken electronegativity (*χ*) and chemical potential (*µ*) further corroborate this picture: *χ* increases monotonically from the hydronium ion complex at N16 (3.4745 eV) through neutral Guanine (5.0004 eV) and the hydroxyl radical adduct at C17 (5.2261 eV) to the dual-attack system (5.3578 eV), while *µ* correspondingly becomes most negative (− 5.3578 eV) for the dual damage. The increasingly negative chemical potential of the stressed species signals a thermodynamic driving force for electron inflow from surrounding biomolecular donors, rationalizing the propensity of the dual-attack adduct to propagate redox reactions along the DNA strand.

The global electrophilicity index (*ω*), integrates both electronegativity and hardness to yield a single measure of the stabilization energy gained upon maximal electron acceptance. Neutral guanine (*ω* = 9.1630 eV) already ranks among moderately strong electrophiles, consistent with its role as a preferential oxidation target in DNA. Hydroxyl radical addition augments this value modestly (*ω* = 11.3348 eV), reflecting the electron-withdrawing character of the newly installed C–O functionality. In sharp contrast, the hydronium ion complex displays a dramatically reduced electrophilicity (*ω* = 2.2055 eV), consistent with its expanded HOMO–LUMO gap and high hardness: isolated protonation at N16 attenuates rather than amplifies electrophilic reactivity. The dual-attack adduct surpasses all other systems by a wide margin, recording *ω* = 34.2618 eV, nearly four times that of the hydroxyl adduct alone and approximately fifteen times that of the hydronium complex. This exceptional electrophilicity index reflects the synergistic combination of a very low hardness (0.4189 eV) with a strongly negative chemical potential (− 5.3578 eV), and provides a quantitative basis for understanding why simultaneous oxidative and acidic attack produces a profoundly reactive damage capable of engaging DNA repair recognition proteins in modes qualitatively different from single-agent damage.

The maximum charge transfer capacity (ΔNmax) quantifies how many electrons the system can accept from an ideal electron donor. Neutral guanine (ΔNmax = 3.6649 e) and the hydroxyl radical adduct (4.3378 e) show moderate electron uptake potential. The hydronium complex shows the lowest uptake capacity (1.2696 e), reinforcing its kinetically protected character under isolated acidic conditions. The dual-attack damage exhibits a striking ΔNmax = 12.7895 e, reflecting its extreme polarizability and capacity to draw electron density from proximal donors such as the phosphate backbone, a conclusion directly corroborated by the large NBO second-order stabilization energies (E₂ up to 53.38 kcal·mol⁻¹) reported in Sect.  3.5.3. The local electrophilic (*ω*⁺) and nucleophilic (*ω*⁻) philicity descriptors follow a similar hierarchy: for the dual-attack complex, *ω*⁺ = 31.6353 eV and *ω*⁻ = 36.9931 eV, values that dwarf those of all other systems. Importantly, the negative difference Δ*ω*± = −5.3578 eV for the dual-attack system (versus − 5.0004, − 5.2261, and − 3.4745 eV for G, the hydroxyl radical adduct, and the hydronium ion complex, respectively) indicates that nucleophilic reactivity slightly dominates over electrophilic reactivity in all four systems, but the absolute magnitude of both components is uniquely amplified in the dual damage, reflecting a globally hyperactivated reactivity state.

The philicity index (PI), provides a composite reactivity metric that simultaneously encodes both electrophilic and nucleophilic capacity. The dual-attack system achieves PI = 81.7862 eV², a value more than eight times that of the hydroxyl radical adduct (9.4081 eV²), approximately 12 times that of neutral guanine (6.7159 eV²), and more than 100 times that of the hydronium complex alone (0.8059 eV²). This exponential amplification of the composite philicity in the dual-attack system underscores the cooperative, synergistic nature of simultaneous oxidative and acidic stress: neither insult alone approaches the reactivity profile generated by their concurrence. The Fukui function fractional index *φ*, measures the electrophilic fraction of the total philicity. Neutral guanine (*φ* = 0.2729) and the hydroxyl radical adduct (0.2305) show similar, moderate electrophilic fractions, while the hydronium complex presents the highest *φ* (0.7877), confirming that its residual reactivity is predominantly electrophilic in character, despite the low absolute magnitude. The dual-attack species exhibits the lowest *φ* (0.0782), indicating that while its absolute electrophilic and nucleophilic powers are both exceptionally high, the nucleophilic component dominates—a balance that may influence which repair proteins recognize and bind the dual damage.

The total dipole moment (TDM) reports on the overall polarity and charge asymmetry of each system. Neutral guanine already bears a substantial dipole moment (12.3083 D), arising from the polar carbonyl group, ring nitrogen atoms, and the phosphate fragment. Hydroxyl radical addition at C17 elevates the TDM to 18.7701 D—the highest in the series—consistent with the introduction of a strongly polarizing C–O bond at an asymmetric position of the purine ring and with the MESP analysis (Sect.  3.2) showing pronounced negative potential redistribution. The hydronium ion complex reduces TDM to 9.2749 D, reflecting partial charge neutralization at the protonation site and the symmetric hydrogen-bond network that delocalizes the proton-induced dipole contribution. The dual-attack system recovers a high TDM of 18.3259 D, close to that of the hydroxyl radical adduct, signifying that the combined damage is highly polarized and generates strong local electric fields within the DNA environment. Elevated dipole moments are relevant to DNA stability in solvent and to the binding affinities of repair proteins: highly polar damage are expected to interact more strongly with the polar active sites of base excision repair (BER) and nucleotide excision repair (NER) enzymes, potentially altering recognition kinetics and contributing to the biological persistence of the dual damage.

Taken together, the quantum reactivity descriptor profile reveals a clear and mechanistically coherent trend across the four systems. These findings provide a rigorous quantum chemical rationale for the heightened biological persistence, altered repair recognition, and enhanced mutagenic potential of the dual oxidative/acidic damage predicted computationally and inferred from radiobiological observations of complex DNA damage in tumor microenvironments.

#### Topological (QTAIM) study of neutral guanine–hydroxyl radical and hydronium ion complexes

Following geometry optimization, Quantum Theory of Atoms in Molecules (QTAIM) analysis was performed on all optimized structures to identify bond critical points (BCPs) and evaluate electronic properties including electron density ρ(r), Lagrangian kinetic energy G(r), potential energy density V(r), energy density H(r), Laplacian of electron density ∇²ρ(r), and ellipticity ε. The QTAIM calculations were used to assess the electronic changes upon hydroxyl radical attachment to the guanine DNA single-strand structure.

##### First complex bond critical point analysis

**Fig. 10:**
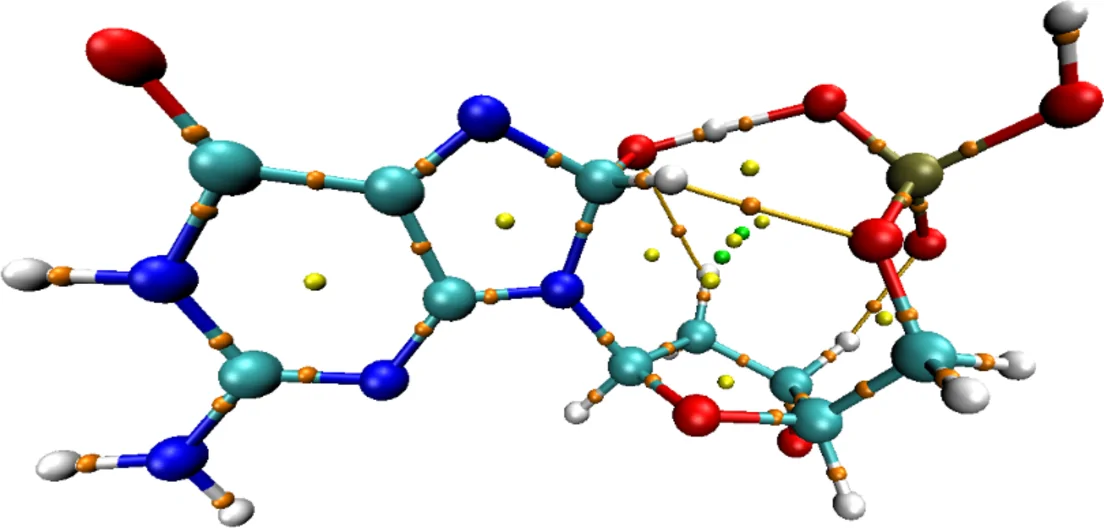
QTAIM analysis of guanine–•OH complex with attack at C17.

Bond critical point analysis of the first guanine DNA single-strand hydroxyl radical complex reveals one primary covalent bond and four hydrogen bonding interactions following hydroxyl radical (•OH) [O(36)-H(37)] attack at the C(17) position (Table S3, Fig. 10). The dominant intermolecular covalent bond at CP46 [O(36)-C(17)] exhibits substantial electron density (ρ = 0.325 a.u.), strongly negative energy characteristics (V(r) = -0.853 a.u., H(r) = -0.526 a.u.), and negative Laplacian (∇²ρ = -0.794 a.u.), confirming stable C—O bond formation with low ellipticity (ε = 0.08) characteristic of σ-type bonds. Intermolecular hydrogen bonds between the hydroxyl radical and guanine occur at CP39 [O (2)-H (37)] with moderate electron density (ρ = 0.096 a.u.) and stabilizing energy (H(r) = -0.04 a.u.), and at CP56 [O (36)-H (25)] with weaker characteristics. Intramolecular hydrogen bonds within the guanine structure at CP55 [H(32)-O(4)] and CP73 [O(3)-H(26)] maintain nucleotide conformational stability during radical attachment. All hydrogen bonds display positive Laplacian values (∇²ρ = 0.047–0.119 a.u.) and moderate ellipticity (ε = 0.013–0.15), confirming their closed-shell, electrostatic nature. This analysis demonstrates irreversible hydroxyl radical covalent attachment supported by both intermolecular radical-guanine and intramolecular guanine stabilization interactions.

##### Second complex bond critical point analysis

**Fig. 11:**
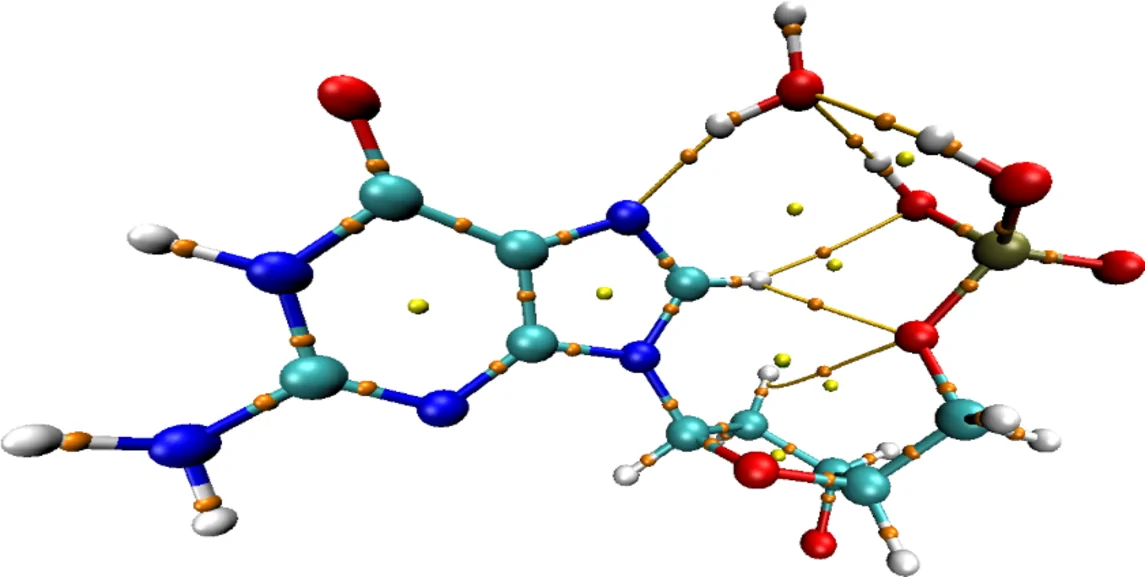
QTAIM analysis of guanine–H₃O⁺ complex with attack at N16 (Second complex).

Bond critical point (BCP) analysis of the second guanine–hydronium ion complex, involving H₃O⁺ [H(37)O(36)–H(38)H(39)] attack at N(16), reveals a complex interplay of covalent and non-covalent interactions (Table S4, Fig. 11). A total of seven distinct critical points were identified, reflecting the diversity of bonding environments within the system. The most prominent feature is a covalent O–H bond at CP90 between O (2) and H (37), supported by high electron density (ρ = 0.33 a.u.), a strongly negative potential energy density (V(r) = − 0.618 a.u.), and a large negative Laplacian (∇²ρ = − 1.895 a.u.). These parameters, together with minimal ellipticity (ε = 0.012), confirm a stable, cylindrically symmetric O–H bond with significant energy stabilization (H(r) = − 0.546 a.u.). Within the hydronium ion itself, an intramolecular hydrogen bond is observed at CP92 between H(37) and O(36), showing moderate electron density (ρ = 0.039 a.u.) and near-zero energy density (H(r) = − 0.001 a.u.), indicative of weak stabilization. The guanine framework maintains its overall integrity through three internal weak contacts: CP65 [H(25)–O(4)], CP72 [O(4)–H(32)], and CP81 [H(32)–O(2)]. These interactions exhibit low electron densities (ρ = 0.008–0.015 a.u.) and slightly positive energy densities (H(r) ≈ 0.001 a.u.), consistent with van der Waals-type interactions. At the guanine–hydronium interface, two intermolecular hydrogen bonds contribute to stabilization: CP87 [N(16)–H(39)] with notable electron density (ρ = 0.047 a.u., H(r) = − 0.003 a.u.), and CP89 [H(35)–O(36)] showing moderate hydrogen bonding features (ρ = 0.034 a.u., H(r) = − 0.001 a.u.). For all non-covalent contacts, the positive Laplacian values (∇²ρ = 0.029–0.112 a.u.) confirm their closed-shell, electrostatic nature, while ellipticity values (ε = 0.041–1.276) indicate varying degrees of anisotropy and charge distribution distortion. Overall, this topological characterization demonstrates that hydronium ion attachment occurs via covalent bond formation, reinforced by a supportive hydrogen-bonding network that preserves the structural integrity of guanine.

##### Third complex bond critical point analysis

**Fig. 12:**
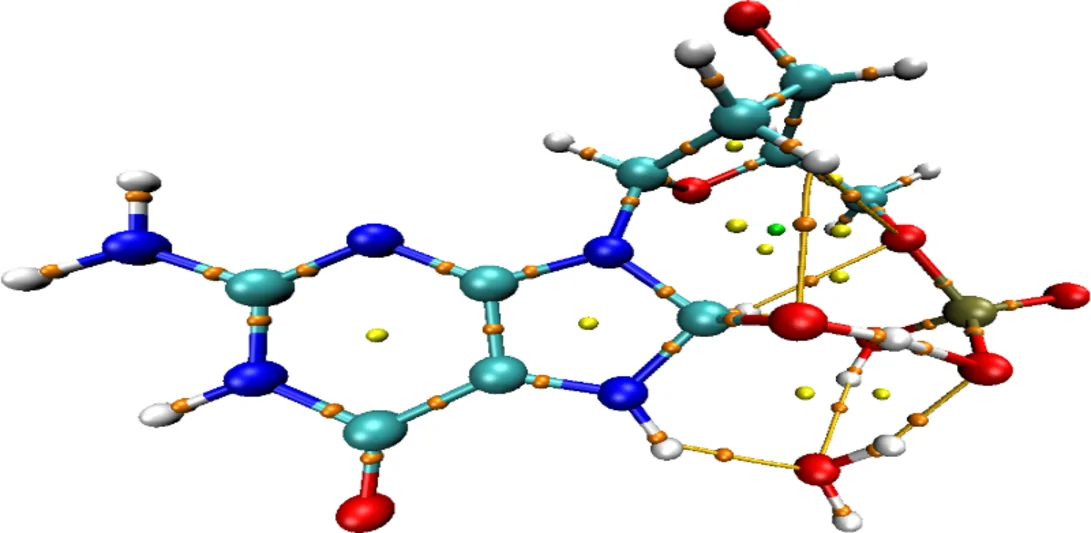
QTAIM analysis of guanine complex with simultaneous •OH and H₃O⁺ attacks at C17 and N16, respectively (Third complex).

The bond critical point (BCP) analysis of the third complex system, representing simultaneous dual attacks by a hydroxyl radical at C(17) [O(40)–H(41)] and a hydronium ion at N(16) [H(37)O(36)–H(38)H(39)], reveals a sophisticated interaction network that mimics a biologically relevant oxidative and acidic stress environment (Table S5, Fig. 12). Topological examination of the electron density distribution identifies ten distinct critical points, comprising two covalent bonds, one intramolecular hydroxyl hydrogen bond, two intramolecular guanine hydrogen bonds, and five intermolecular hydrogen bonds linking guanine with the hydroxyl radical and hydronium ion. The covalent interactions [39(H)–16(N) and 40(O)–17(C)] are defined by high electron densities (ρ = 0.321 and 0.312 a.u.) and strongly negative energy densities (H(r) = − 0.481 and − 0.490 a.u.), confirming irreversible bond formation. Their negative Laplacian values (∇²ρ = − 1.761 and − 0.822 a.u.) indicate localized charge concentration along the internuclear axes, typical of shared-shell covalent bonds. In contrast, the hydrogen-bonding network exhibits electron densities between 0.009 and 0.077 a.u., with energy densities near zero or slightly positive, consistent with closed-shell electrostatic interactions. The five intermolecular hydrogen bonds [37(H)–2(O), 36(O)–35(H), 2(O)–41(H), 40(O)–25(H), and 4(O)–25(H)] display moderate-to-weak binding strengths, whereas the intramolecular guanine hydrogen bonds [34(O)–32(H) and 32(H)–4(O)] are especially weak (ρ = 0.009 and 0.015 a.u.), implying minimal disruption of the intrinsic base-pairing geometry. Ellipticity analysis shows generally low values across most interactions, with the exception of the weak intramolecular contact [34(O)–32(H)] (ε = 2.178), indicating localized anisotropy and deviation from cylindrical charge distribution. Taken together, these results provide quantitative evidence of stable covalent adduct formation at C(17) and N(16), reinforced by an extensive hydrogen-bonding coordination sphere. This dual-attack scenario underscores the molecular basis of oxidative and acid-induced DNA damage at the electronic structure level, where covalent damage formation is complemented by a stabilizing secondary hydrogen-bond network.

#### Non-covalent interaction (NCI) analysis of hydroxyl radical (•OH) and hydronium Ion (H₃O⁺) guanine complexs

**Fig. 13:**
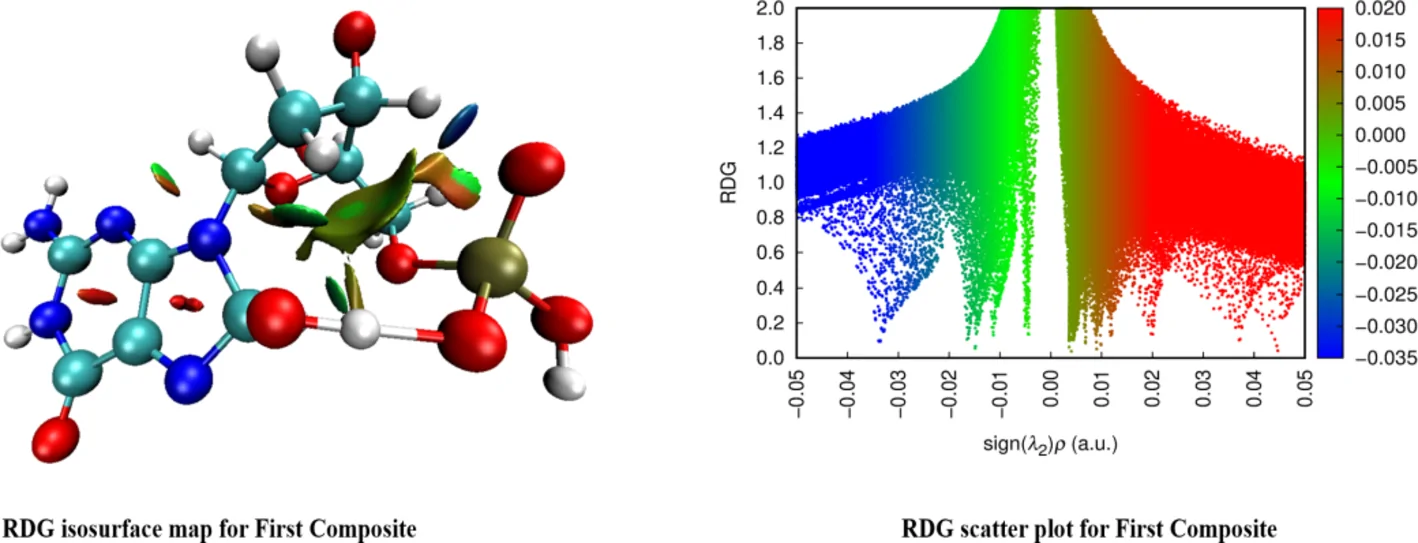
NCI of guanine–•OH complex with attack at C17 (First complex).

**Fig. 14:**
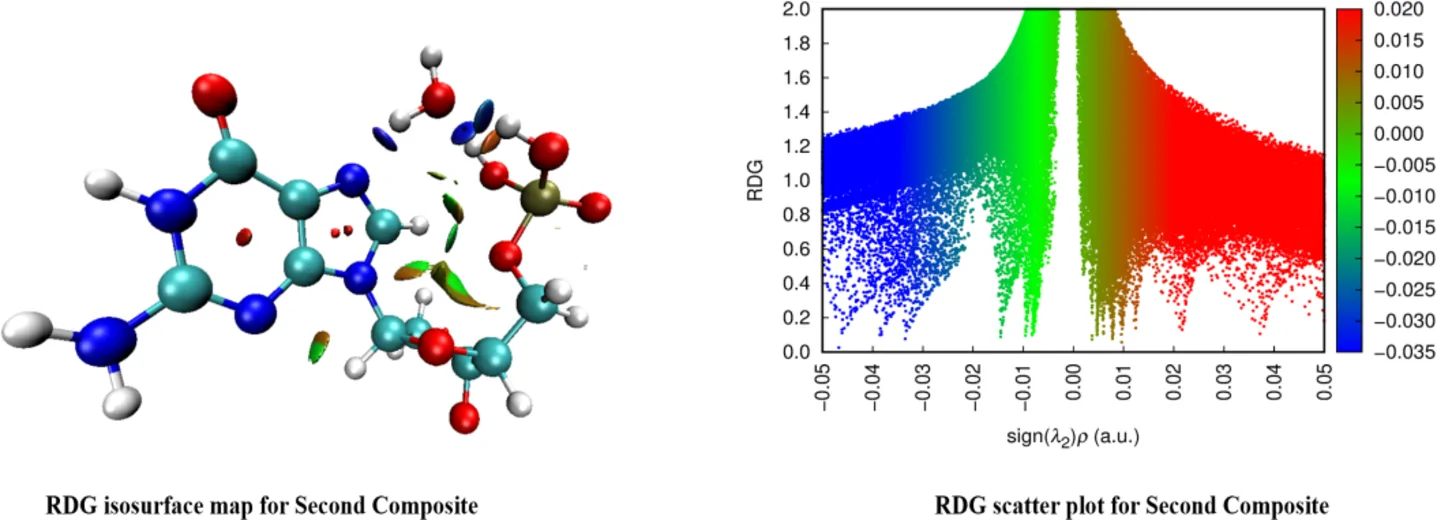
NCI analysis of guanine–H₃O⁺ complex with attack at N16 (Second complex).

**Fig. 15:**
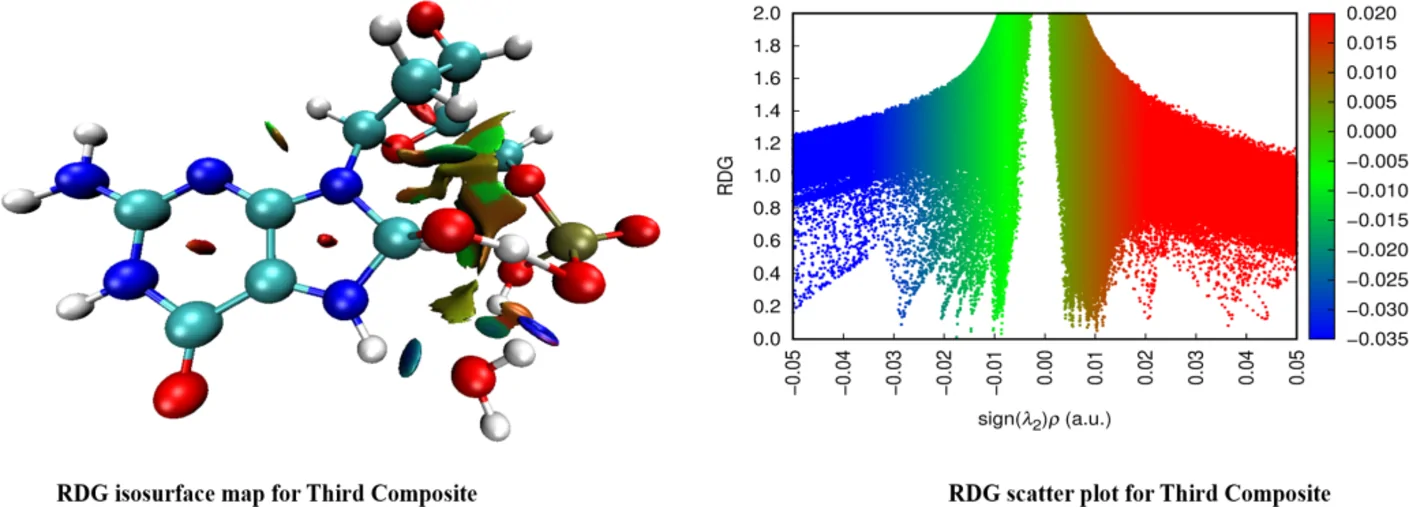
NCI analysis of guanine complex with simultaneous •OH and H₃O⁺ attacks at C17 and N16, respectively (Third complex).

To complement the QTAIM characterization, Non-Covalent Interaction (NCI) analysis was employed to visualize the spatial distribution and relative strength of weak interactions within guanine single-strand complexs involving hydroxyl radical (•OH) and hydronium ion (H₃O⁺). The reduced density gradient (RDG) was mapped against the sign(λ₂)ρ function, enabling differentiation of hydrogen bonding (blue), van der Waals forces (green), and steric repulsion (red). For the hydroxyl radical–guanine complex (Fig. 13), distinct blue RDG isosurfaces were localized at the radical-base interface, confirming intermolecular hydrogen bonds at CP39 [O(2)-H(37)] and CP56 [O(36)-H(25)], as well as intramolecular interactions at CP55 [H(32)-O(4)] and CP73 [O(3)-H(26)], consistent with QTAIM findings. Peripheral green isosurfaces further indicate dispersion-dominated contacts contributing to structural stabilization. In the hydronium ion–guanine complex (Fig. 14), extensive blue surfaces clustered around the N(16) binding site highlight the dominance of strong hydrogen bonding interactions, with greater density and intensity compared to the hydroxyl radical system. This is in agreement with the higher electronegativity and hydrogen-bonding capacity of the N(16) locus, supporting QTAIM evidence of enhanced stabilization. The dual-attack complex (hydroxyl radical at C(17) and hydronium ion at N(16), Fig. 15) displayed multiple blue regions surrounding both attachment points, reflecting cooperative hydrogen bonding within the adduct network. Red isosurfaces were also observed, signifying steric crowding and local destabilization arising from the proximity of the dual damage. Scatter plot analysis further demonstrated that hydrogen bond strength follows the order: dual attack (•OH + H₃O⁺) > hydronium ion at N(16) > hydroxyl radical at C(17). This hierarchy closely correlates with QTAIM-derived electron density and energy density values, providing consistent evidence of relative stabilization trends. Collectively, the NCI analysis not only corroborates QTAIM predictions of covalent and hydrogen bonding interactions but also reveals the presence of dispersion contacts and steric constraints. These findings highlight how oxidative (•OH) and acidic (H₃O⁺) stresses synergistically enhance local binding networks while imposing conformational strain, offering deeper insight into the molecular determinants of DNA damage under complex microenvironmental conditions.

#### Natural bond orbital (NBO) analysis of hydroxyl radical (•OH) and hydronium ion (H₃O⁺) guanine complexes

Natural bond orbital (NBO) analysis provides valuable insights into the electronic structure and bonding interactions in molecular systems. Based on **Fock matrix** theory, NBO evaluation quantifies intramolecular interactions between filled (donor) and empty (acceptor) orbitals. The stabilization energy E2 obtained from NBO analysis measures the strength of these donor-acceptor interactions, arising from electron density transfer from occupied to unoccupied orbitals. This parameter serves as an indicator of orbital delocalization and electronic stabilization within the molecule [43].

##### First complex natural bond orbital (NBO) analysis

The NBO analysis of the guanine hydroxyl radical complex reveals a network of stabilizing donor acceptor interactions centered on the hydroxyl group [O(36)-H(37)] attached at the C(17) position (Table S6). The most significant stabilization originates from the phosphate oxygen, where the BD(2) P(1)-O(2) → σ*(O36–H37) delocalization contributes 31.80 kcal·mol⁻¹ (ΔE = 0.78 a.u., Fij = 0.199) a.u., characteristic of a strong charge-assisted hydrogen bond between the phosphate oxygen and the hydroxyl group. This effect is further reinforced by lone-pair donation from O(2) [LP(1) O(2) → σ*(O36–H37)], with an additional stabilization energy of 9.90 kcal·mol⁻¹ (ΔE = 1.02 a.u., Fij = 0.129). Beyond hydrogen bonding, oxygen O(36) engages in hyperconjugative interactions with the guanine backbone. Specifically, LP(2) O(36) → σ*(N16-C17) and LP(2) O(36) → σ*(C17–N18) provide stabilization energies of 6.35 kcal·mol⁻¹ and 9.48 kcal·mol⁻¹, respectively, facilitated by small energy gaps (0.58 − 0.49 a.u.) and substantial Fock matrix couplings, confirming efficient n→σ* donation into adjacent antibonding orbitals. Additional orbital mixing is evident from BD*(1) P(1)-O(2) → σ*(O36-H37) (5.29 kcal·mol⁻¹, ΔE = 0.05 a.u.) and reciprocal σ*(O36-H37) → σ*(C17-H32) (8.83 kcal·mol⁻¹, ΔE = 0.02 a.u.) couplings, reflecting σ*–σ* interaction pathways within the extended hydrogen-bond framework. Taken together, these results establish phosphate oxygen O(2) as the dominant electron donor driving hydrogen-bond-assisted stabilization, while O(36) operates as a multifunctional hub that bridges the hydrogen bond network with the guanine C(17) framework via hyperconjugative and σ*–σ* pathways. This dual role highlights how radical-induced modifications can couple local hydrogen bonding to broader electronic redistribution within the DNA strand.

##### Second complex natural bond orbital (NBO) analysis

The NBO analysis of the guanine–hydronium ion complex reveals a set of four dominant donor–acceptor interactions that define the electronic stabilization at the N(16) attachment site (Table S7). Both N(16) and O(36) act as major electron-donor centers, contributing to the formation of an extensive hydrogen-bond-assisted hyperconjugation network. The strongest stabilization pathway arises from the lone pair on N(16) donating into the σ*(O36-H39) antibonding orbital, with a stabilization energy of 13.97 kcal·mol⁻¹ (ΔE = 0.82 a.u., Fij = 0.137). This pronounced n→σ* interaction couples the guanine nitrogen directly with the hydronium proton, representing the primary mechanism of covalent and electrostatic stabilization. Oxygen O(36) functions as a versatile secondary donor. Both LP(1) O(36) → σ*(O34–H35) and LP(2) O(36) → σ*(O34-H35) provide moderate stabilization energies of 4.38 kcal·mol⁻¹ and 4.70 kcal·mol⁻¹, respectively, reflecting charge-assisted reinforcement of the adjacent O34–H35 framework. Furthermore, LP(2) O(36) donates into σ*(O2–H37) with a stabilization energy of 8.97 kcal·mol⁻¹ (ΔE = 1.04 a.u., Fij = 0.122), effectively bridging the hydronium ion and the phosphate-associated O(2) site. Collectively, these donor–acceptor interactions establish a dual stabilization scheme: N(16) provides the dominant n→σ* hyperconjugation directly into the O36–H39 bond, while O(36) serves as a multifunctional hub, simultaneously reinforcing intramolecular hydrogen bonds and linking the hydronium ion to the guanine backbone and phosphate framework. This cooperative electronic redistribution highlights how hydronium ion attachment at N(16) promotes both localized and extended stabilization within the DNA strand.

##### Third complex natural bond orbital (NBO) analysis

The NBO analysis of the dual-attack complex (•OH at C17 and H₃O⁺ at N16; Table S8) identifies a small number of dominant charge-transfer pathways that collectively stabilize the reaction center and couple the damage to the DNA backbone. Two classes of interactions emerge as most significant. First, charge-assisted hydrogen-bond (CAHB) donations from the phosphate oxygen O(2) provide the largest single stabilizing contributions and act as directional electronic bridges to both attack sites. Notably, LP(3) O(2) → σ*(O40-H41) yields E(2) = 53.38 kcal·mol⁻¹ (ΔE = 0.75 a.u., Fij = 0.182), while LP(2) O(2) → σ*(O36-H37) gives E(2) = 29.13 kcal·mol⁻¹ (ΔE = 0.76 a.u., Fij = 0.136). These large E(2) values indicate very efficient, charge-assisted hyperconjugative stabilization that couples the phosphate with both the hydroxyl and hydronium adducts. Second, n→σ hyperconjugation from the attacking oxygen (O(40)) into the nascent C–N σ framework** at C17 provides substantial local delocalization of electron density away from the reactive center. Specifically, LP(2) O(40) → σ*(N16–C17) and LP(2) O(40) → σ*(C17-N18) contribute 15.66 kcal·mol⁻¹ and 8.37 kcal·mol⁻¹, respectively (ΔE ≈ 0.58 a.u., Fij ≈ 0.087/0.063). These interactions help disperse radical character across adjacent σ* acceptors and mitigate localized electronic strain at C17. Supporting, but smaller, contributions arise from the aromatic backbone: BD(2) C15-N16 and BD(2) N18-C19 → σ*(C17-O40) each contribute ~ 5.8 kcal·mol⁻¹ (ΔE ≈ 0.73–0.74 a.u.), consistent with modest, symmetric π-donation flanking the modified carbon. Additional pathways of lower magnitude include LP(1) O(40) → RY*(2) C(17) (4.04 kcal·mol⁻¹, large ΔE = 1.50 a.u.) and LP(1) O(40) → σ*(C17-H32) (5.49 kcal·mol⁻¹), which likely reflect charge flow into diffuse Rydberg/σ* acceptors proximal to the reactive site. Several entries where antibonding orbitals act as donors (for example, σ*(P1–O2) → σ*(O40-H41) and σ*(O40-H41) → σ*(C17-H32), E(2) ≈ 5–6 kcal·mol⁻¹ with very small ΔE = 0.04–0.07 a.u.) indicate near-degenerate orbital mixing within the extended hydrogen-bond network rather than classical donor→acceptor delocalization; these should therefore be interpreted as signatures of reciprocal σ*–σ* coupling rather than simple one-way charge transfer. At the N(16) site, LP(1) O(36) → σ*(O34–H35) (5.14 kcal·mol⁻¹) contributes to an extended hydrogen-bond chain that further stabilizes the hydronium adduct. In summary, the NBO results portray O(2) as the principal electron donor, mediating large CAHB-driven stabilization to both adduct sites, while O(40) supplies effective n→σ* delocalization into the C17–N framework. The combined action of these pathways disperses the radical-induced perturbation, creates an energetically favored coordination sphere around the damage, and thus stabilizes the dual-attack complex. These electronic couplings rationalize how phosphate-mediated charge assistance and oxygen-centered hyperconjugation cooperate to render simultaneous oxidative and acidic attacks on guanine both structurally and electronically viable.

## Recommendations and future directions

To enhance the predictive power and translational impact of this work, we propose a systematic multiscale framework. Key priorities include:

### Advanced Solvation and System Modeling

Employ hybrid explicit/implicit solvent models and QM/MM simulations of full DNA duplexes to accurately capture solvation dynamics, base stacking, and long-range electrostatic effects. Modeling the syn glycosidic conformation to assess its influence on frontier orbital character.

### Electronic Structure and Benchmarking

Validate key species with higher-level methods and contemporary, dispersion-corrected functionals. Implement multireference diagnostics for open-shell intermediates to ensure reliability.

### Increased Biological Fidelity

Model reactions within realistic reactive oxygen/nitrogen species (RONS) mixtures and variable stoichiometries to better emulate pathological microenvironments. Bridge atomistic results to mesoscale radiolysis Monte Carlo simulations (e.g., Geant4-DNA, TOPAS-nBio) to predict damage spectra under different irradiation conditions.

### Experimental Collaboration and Reproducibility

Design targeted experiments (HPLC-MS/MS, ESR, NMR) to validate computational predictions. To ensure reproducibility, all optimized geometries, input files, and analysis scripts should be deposited in a public repository with comprehensive metadata. Implementing this strategy will transform our findings into quantitative, testable predictions, thereby accelerating translation to radiobiology and mutagenesis studies.

## Conclusion

The quantum-chemical study demonstrates that when both oxidative stress (•OH) and acidic stress (H_3_O^+^) simultaneously attack guanine they create a special complex damage which remains highly stable and differs from all types of damage found in traditional research. The results show that •OH primarily creates a C–O bond with C17 (the standard C8 position) which results in extensive π-conjugation loss while H_3_O^+^ applies protons to N16 (the standard N7 position) which results in a stabilized hydrogen-bonded structure. The combined attacks create the strongest electron-density changes in this research which show internal steric strain growth and total dipole moment (TDM) values that demonstrate a highly reactive and polarized condition.

The QTAIM and NCI topological analysis shows that the dual-stress system obtains energetic stability through its complex system of hydrogen bonds which connects different molecular parts and through its phosphate oxygen atoms which improve hyperconjugation. The results of HOMO–LUMO analysis show that the energy gap between the two states has decreased which leads to higher chemical reactivity and the movement of electronic states that cause additional molecular damage. MESP maps identify critical shifts in the nucleophilic and electrophilic regions, highlighting the specific sites where guanine’s chemical identity is most compromised. This electronic and structural characterization provides a detailed reference for the intrinsic reactivity of the complex damage. The study results establish a mechanistic framework detailing how coexisting acidic and oxidative stressors jointly reshape guanine’s electronic structure and bonding topology at the molecular level of DNA damage within the boundaries of the present computational model.

## Data Availability

All data supporting the findings of this study are available from the corresponding author on reasonable request.
